# Bone reconstruction with modified Masquelet technique in open distal femoral fractures: a case series

**DOI:** 10.1186/s12891-023-07091-5

**Published:** 2024-01-02

**Authors:** Seyed Hadi Kalantar, Hana Saffar, Amir Human Hoveidaei

**Affiliations:** 1 Joint Reconstruction Research Center, IKHC, Tehran University of Medical Science, Tehran, Iran; 2 Cancer Institute, IKHC, Tehran University of Medical Science, Tehran, Iran; 3 Sports Medicine Research Center, Tehran University of Medical Sciences, Tehran, Iran

**Keywords:** Bone defect, Bone loss, Induced membrane, Open femoral fracture

## Abstract

**Background:**

Large bone defects require complex treatment, multidisciplinary resources, and expert input, with surgical procedures ranging from reconstruction and salvage to amputation. The aim of this study was to provide the results of a case series of open comminuted intra-articular distal femoral fractures with significant bone loss that were managed by early fixation using anatomical plates and a modified Masquelet technique with the addition of surgical propylene mesh.

**Methods:**

This retrospective study included all patients referred to our institution with OTA/AO C3 distal femur open fractures and meta-diaphyseal large bone loss between April 2019 and February 2021. We treated the fractures with irrigation and debridement, acute primary screw and plate fixation in the second look operation, and Masquelet method using shell-shaped antibiotic beads supplemented by propylene surgical mesh to keep the cements in place. The second step of the procedure was conducted six to eight weeks later with bone grafting and mesh augmentation to contain bone grafts. Surprisingly, hard callus formation was observed in all patients at the time of the second stage of Masquelet procedure.

**Results:**

All five patients’ articular and meta-diaphyseal fractures with bone loss healed without major complications. The average union time was 159 days. The mean knee range of motion was 5–95 degrees. The average Lower Extremity Functional Score (LEFS) was 49 out of 80.

**Conclusions:**

Combination of early plate fixation and the modified Masquelet technique with polypropylene mesh is an effective method for managing large bone defects in open intra-articular distal femoral fractures with bone loss, resulting in shorter union time possibly associated with the callus formation process. This technique may also be applicable to the management of other similar fractures specially in low-income and developing areas.

## Background

Distal femur fractures are the second most frequent fracture type of femur fractures with an annual incidence rate of 8.7 per 100,000 [8, 16]. Among these fractures, the management of intra-articular AO/OTA type 33-C3 femoral fractures of the distal femur are considered to be notably challenging [4]. This management becomes more challenging in cases with open fractures along with a large bone defect [12]. Bone defects of the limbs are defined as a bone loss without any new bone formations or areas where regenerated new bone accounts for less than 10% of the bone defect at the extremities. They are typically caused by trauma, chronic osteomyelitis, or malignant tumor resection [36]. Large bone defects persist as a difficult problem, especially in defects larger than 5 cm where much is still unknown regarding their outcomes, necessitating complex treatment, extensive multidisciplinary resources and expert input [11, 26].

Surgical procedures ranging from reconstruction and salvage to amputation have all been mentioned in the literature to manage large bone defects. These methods, as well as allografts, direct cancellous autologous bone grafting, and vascularized fibular bone graft, have all been used in previous studies [17, 23]. The “induced membrane” method for bone reconstruction is a recent development that has piqued the interest of clinicians and basic scientists after 2000 when it was introduced by Masquelet et al. [22].

There are two stages to the Masquelet method; installation of a cement spacer, massive bone graft, initial debridement, soft-tissue restoration by flaps if necessary, and insertion of polymethylmethacrylate (PMMA) cement spacer are all included in the first stage. At least six to eight weeks after the first stage, the second stage is performed. When the spacer is removed, the membrane is filled with autografts of cancellous tissue [22]. Fragment stabilization during this induced membrane procedure is needed and Masquelet et al. recommended nailing in large bone defects [21]; however, nailing is not considered a proper method for C3 distal femoral fractures with articular comminution. Previous studies has reported instability and poor outcomes of these cases following the intramedullary nailing fixation and have recommended plate and screw fixation instead [10, 14]. On the other hand, biofilm formation is a notable disadvantage of fixations with plate [15]; This can result in delayed healing, leading to permanent functional loss, or in severe cases, necessitate amputation of the affected limb [28]. We modified the Masquelet technique by using polypropylene mesh augmentation instead of intramedullary nailing to stabilize the cement beads during the first stage of Masquelet procedure.

In this paper, we present the clinical and radiological outcomes, as well as some interesting findings, of open distal femoral fractures treated early with anatomical plates and the modified Masquelet technique with polypropylene mesh augmentation.

## Methods

### Patients characteristics and study design

We retrospectively evaluated five patients who were referred to our department between December 2019 and March 2021 with open distal femoral fractures AO/OTA 33-C3 and significant bone loss.

Four of these patients sustained a type 3 A Gastillo-Anderson open fracture of the distal femur, while one patient exhibited a type 3 C fracture. Two of the patients were smokers. Patients’ ages ranged between 28 and 58, with a mean of 43.6 years. Other than being smokers in two patients, none of them had other comorbid conditions. After initial debridement and limb length restoration, all of these patients had a segmental bone loss on the meta-diaphyseal portion of the distal femur ranging from 7 to 12 cm with a mean of 9.4 cm. All patients suffered from severe articular surface comminution of the distal femur. Two patients had ipsilateral tibial fractures, while one patient had an ipsilateral acetabular fracture and a contralateral tibial shaft fracture. The remaining three patients had no associated limb fractures. Details are shown in Table 1.

**Table 1:** Patient Characteristics, management and follow-up summary

Patient Number	Age and Sex	Gastillo-Anderson Classification	Fixation Device	Double plating	Defect Size in Antero- Posterior View (cm)	Time to Definite Fracture Fixation (Days)	Timing of Bone Graft from initial cementing (Days)	Autograft Harvest Site	Bone Graft Volume (cc)	Polypropylene Mesh Size (cm^2^)	Union Time after Second Surgery (Months)	History of Smoking	Follow Up (Months)	Lower Extremity Functional Score
1	36 ♂	IIIA	Lateral Anatomical, Medial 4.5 mm LCP	Yes	12	10	50	Iliac Crest	70	30	3	No	12	42
2	54 ♂	IIIA	Lateral Anatomical, Anterior 3.5 mm LCP	Yes	8	6	42	Iliac Crest	50	20	2.5	Yes	14	45
3	58 ♂	IIIC	Lateral Anatomical, Medial 4.5 mm LCP	Yes	7	14	56	Iliac Crest & Fibula	50	15	4	Yes	14	58
4	42 ♂	IIIA	Lateral Anatomical plate	No	11	8	60	Iliac Crest	70	30	4	No	13	57
5	28 ♂	IIIA	Lateral Anatomical plate	No	10	4	48	Iliac Crest	60	20	3	No	16	43

LCP: Locking compression plate

### Surgical technique

#### First stage

After placing the patient in a supine position and applying a tourniquet to minimize blood loss (except for the patient with a type 3 C open fracture), we draped the affected limb. Following thorough irrigation, debridement, and removal of devitalized cortical bone and fixing the articular surface fragments with mini headless or larger cancellous screws, then we assessed the segmental defects. In one case involving an open fracture of type 3 C, external fixation was used with femoral-popliteal bypass. In the second surgical session, patients were brought back to the operating room after 48 h for a second look except for the type 3 C case which the second surgery was performed 14 days later with the blessing of the vascular surgery team. In this session, both lower limbs were draped without the use of a tourniquet. The wound was then opened, and as there was no sign of infection and gross contamination, large amounts of dead tissue, or concerns about soft tissue coverage, the limb length, rotation, and alignment were restored using a lateral anatomical Locking compression plate (LCP) with or without an additional medial 4.5 LCP.

**Fig. 1:**
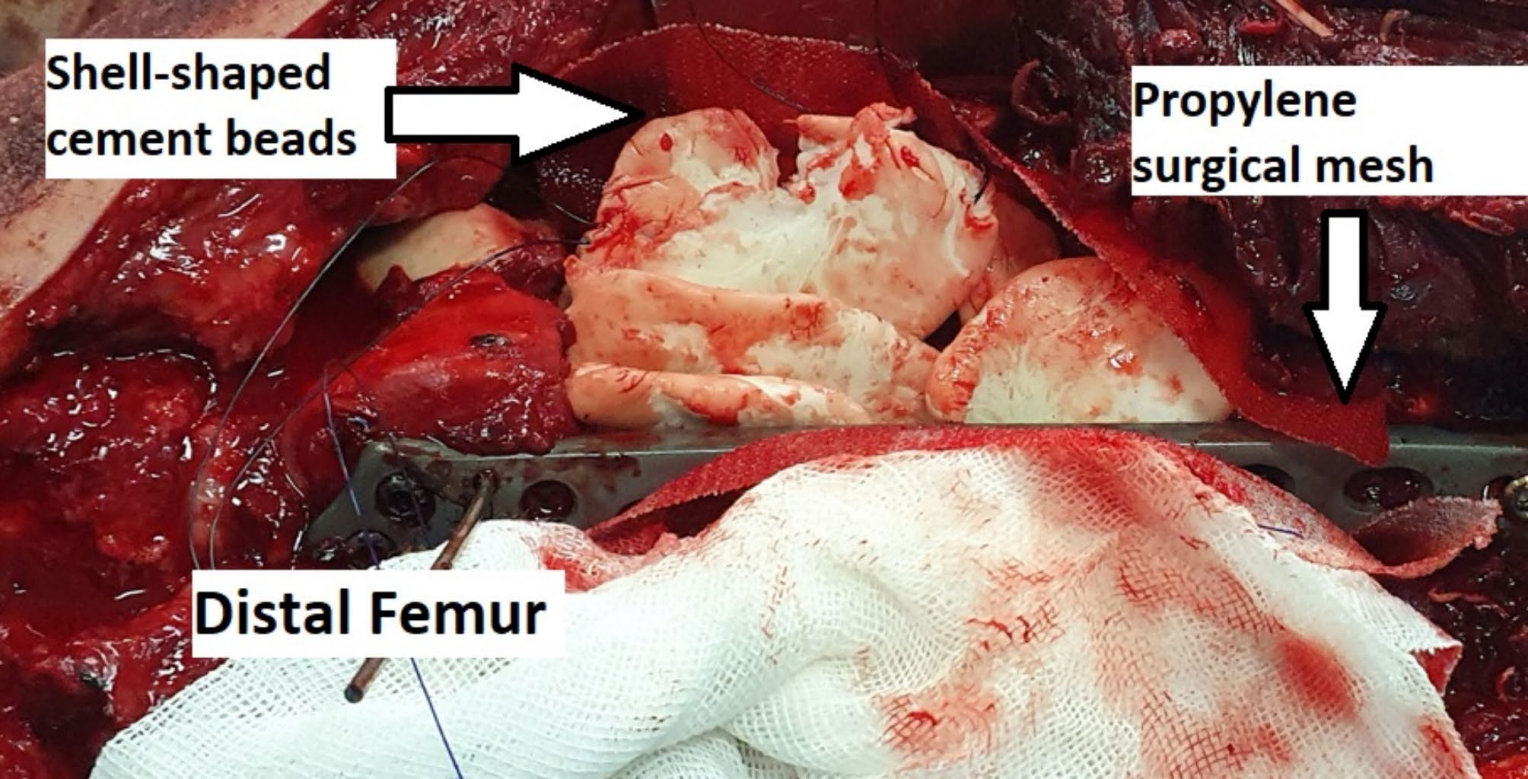
Filling the segmental defect with shell-shaped, antibiotic-impregnated cement beads

**Fig. 2:**
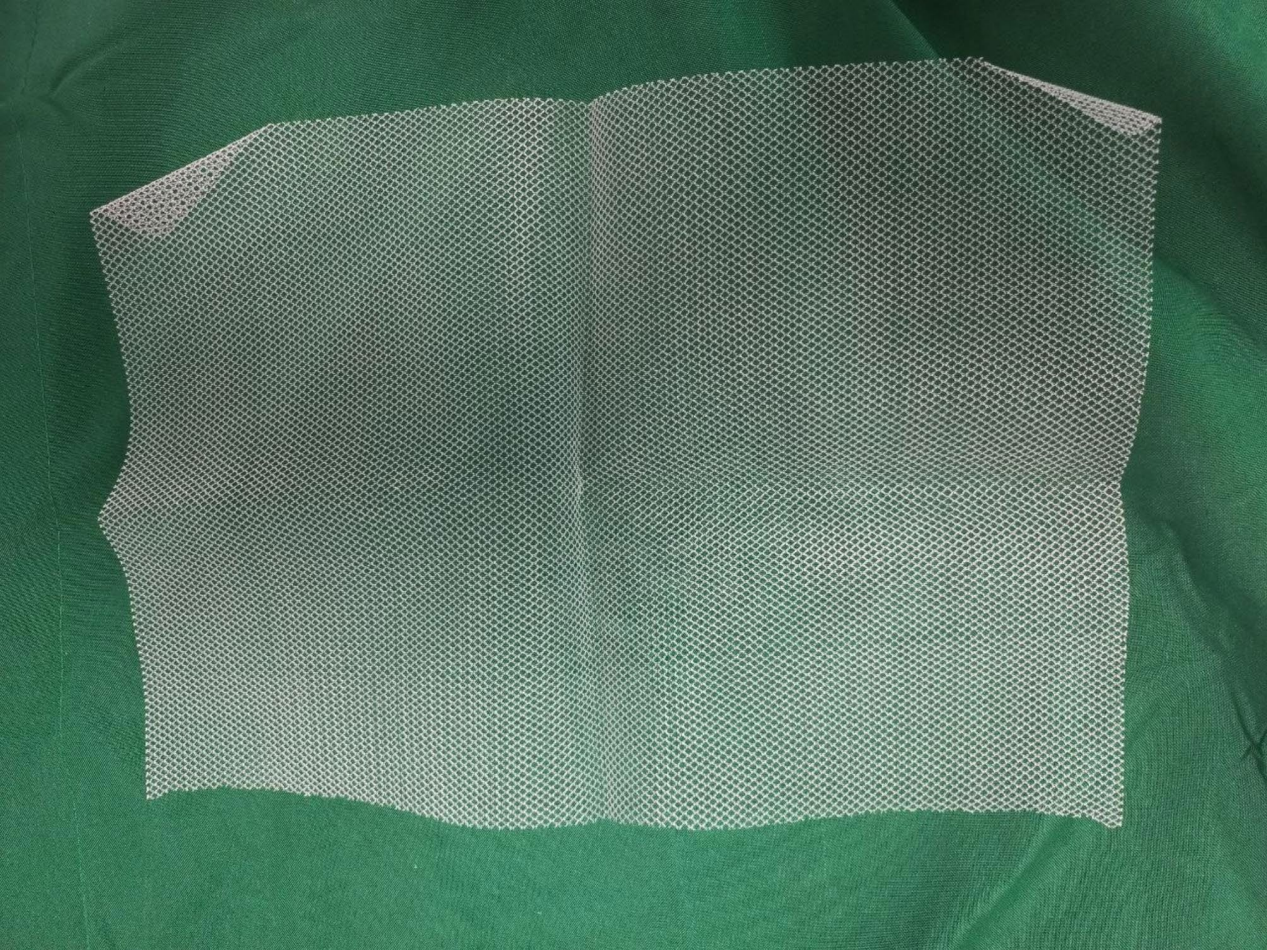
Standard polypropylene surgical mesh

**Fig. 3:**
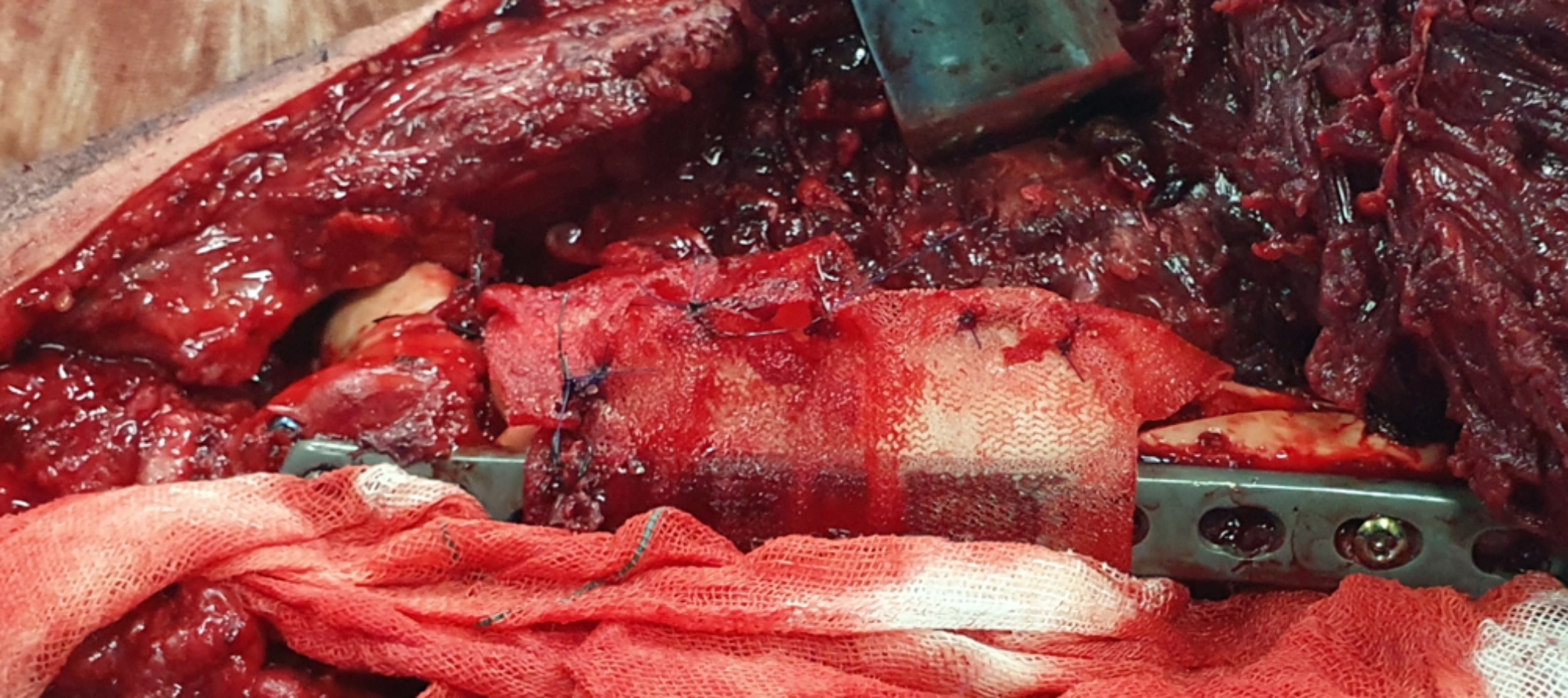
Suturing the mesh to the plate and to itself and layered closure of the wound

To insert the PMMA cement as a spacer, we filled the segmental defect with shell-shaped, antibiotic-impregnated cement beads to not only prevent infection but also induce membrane formation (Fig. 1). We impregnated 2 g of vancomycin into BonOs® R Genta which includes 2.4 g of gentamycin in 40 g of cement powder. We shaped the prepared cements to shell-shaped beads. Then, a standard polypropylene surgical mesh (Fig. 2) [41] was wrapped around the cement beads to contain them and prevent moving and irritating the surrounding muscles during muscle contractions and movements. We used 2/0 round Polydioxanone (PDS) to suture the mesh to the plate and to itself, and we used non-absorbable layered sutures to close the wound (Fig. 3). Patients were encouraged to perform early knee range of motion and were mobilized using non-weight-bearing protocols.

#### Second stage

**Fig. 4:**
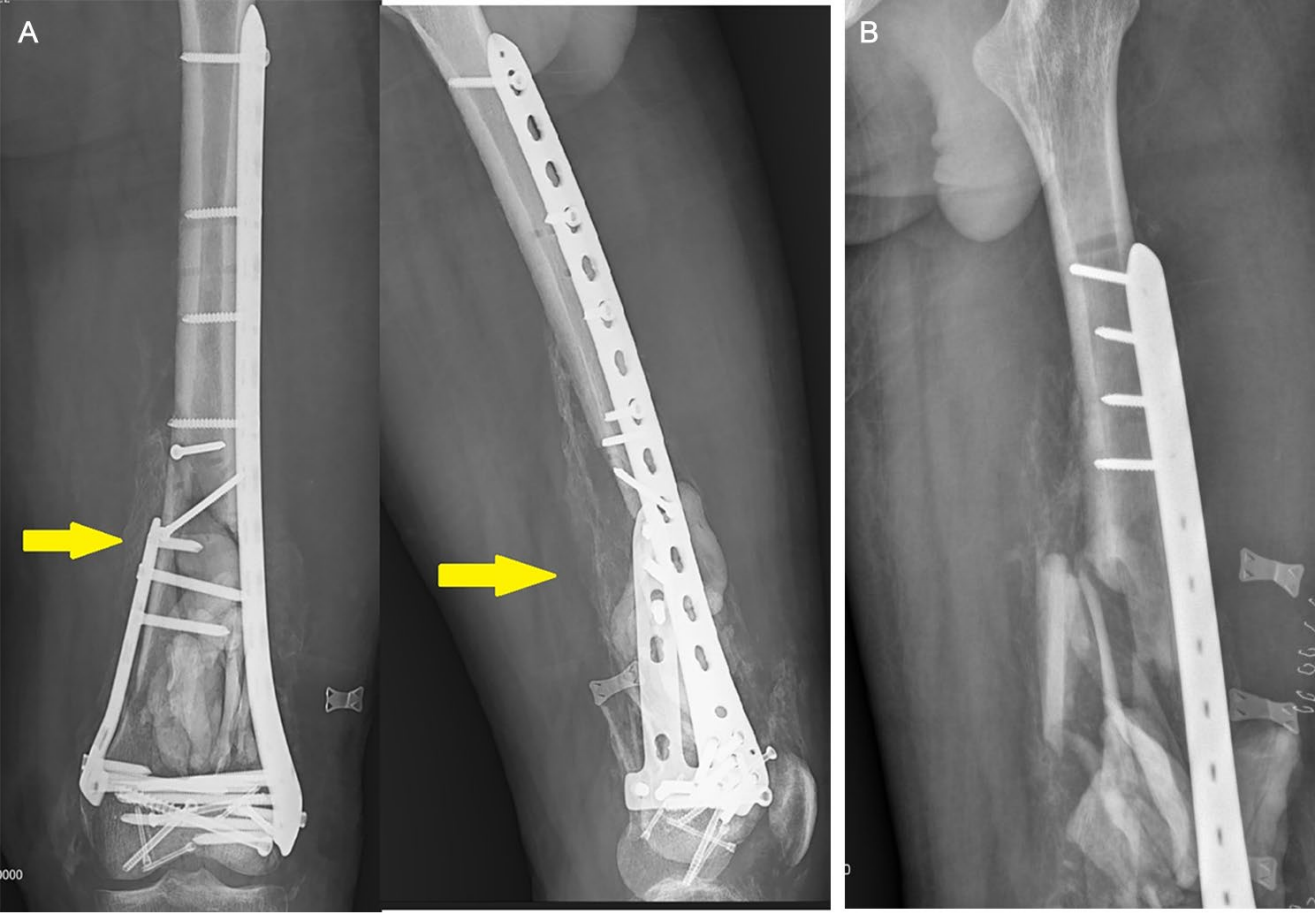
Hard callus formation in X-ray. (**A**) Patient One, (**B**) Patient Two

After confirming that the patients’ serial C-reactive protein (CRP) levels were declining at least in the sixth week after the initial surgery, we admitted them. Before the bone grafting procedure, X-rays of the affected area were taken of all of our patients. Intriguingly, we observed hard callus formation in all patients (Fig. 4).

**Fig. 5:**
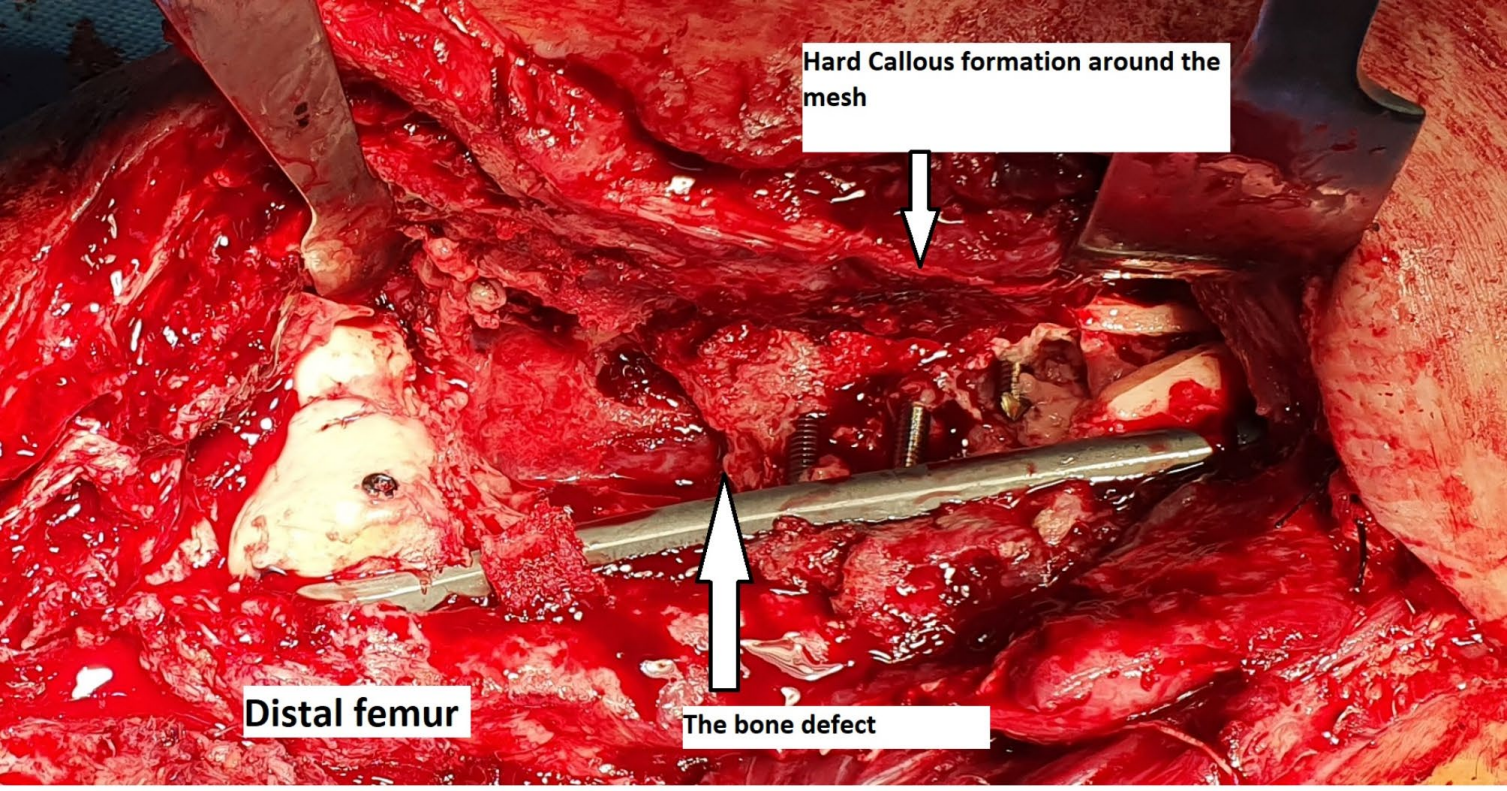
Propylene mesh surrounded by callus formation

**Fig. 6:**
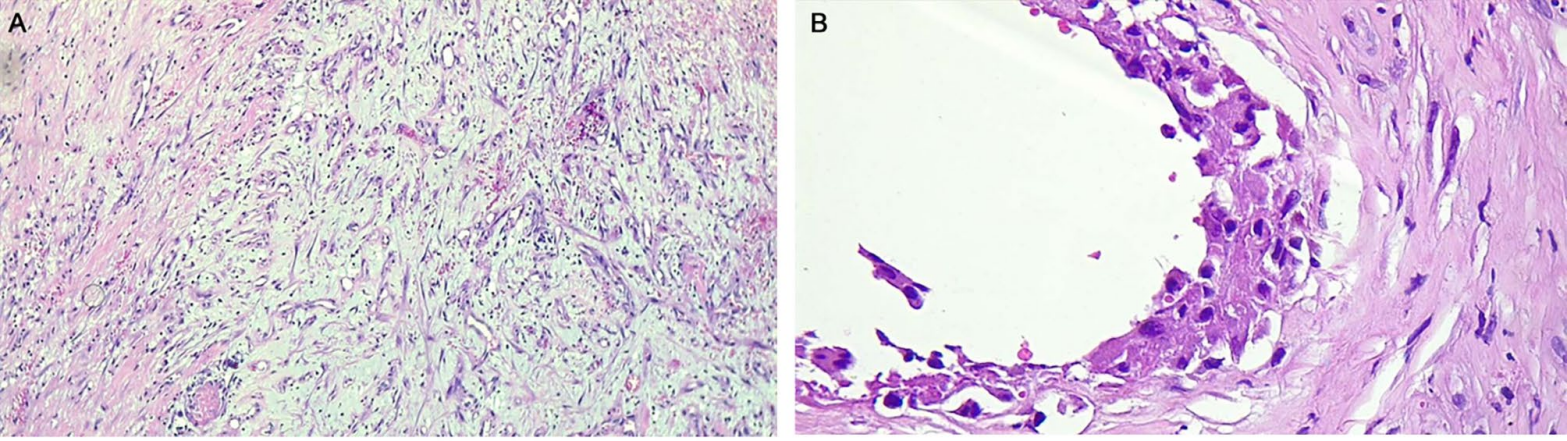
Granulation tissue formation from previous mesh site sections, Hematoxylin & Eosin, x100 (**A**) and x400 (**B**)

During the surgical procedure, after carefully opening the previous incision at the site of bone loss, we encountered a membrane that was considerably tougher than the typically induced membrane described by Masquelet. The polypropylene mesh appeared to be surrounded by callus formation (Fig. 5). We sent a specimen to our pathology laboratory, where the formation of granulation tissue area was reported (Fig. 6), indicating tissue repair as well as foci of fibroblasts, histiocyte aggregation, and foreign body type giant cell reaction.

**Fig. 7:**
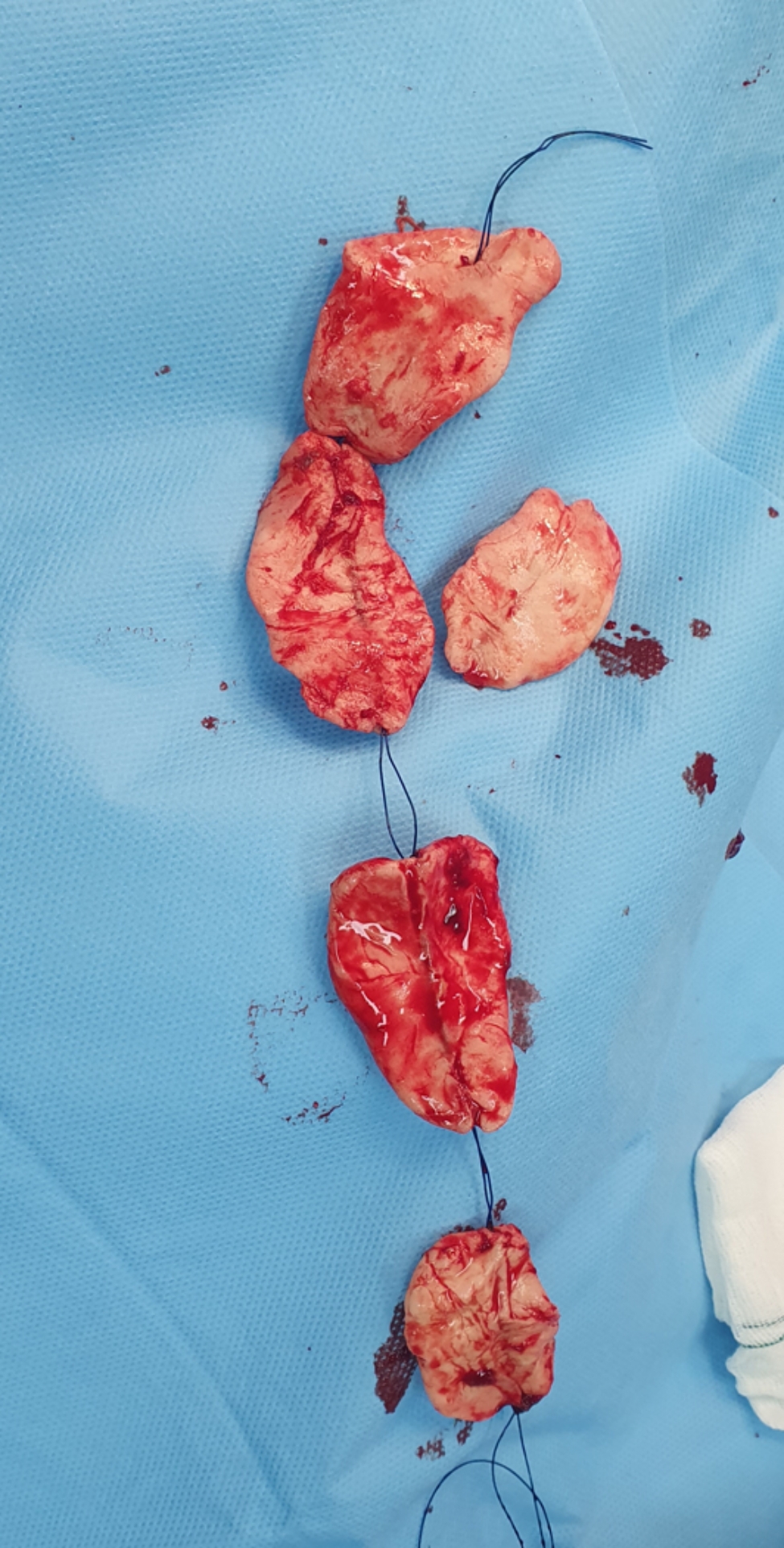
Removing the antibiotic beads

After carefully removing the antibiotic beads (Fig. 7), we drilled and refreshed both sides of the fracture edges before filling in the gaps with a 3 to 1 ratio of autologous iliac cortico-cancellous bone grafts and synthetic bone grafts. However, in one patient (3 C open fracture), we augmented the central part of the defect and graft site with a non-vascularized fibular autograft to increase both stability and union chance. Cortico-cancellous autografts were harvested using osteotomes and curettes in three cases, and acetabular reamers of sizes 36 to 38 from the lateral iliac bone in two other cases where larger volumes of bone graft were required. After filling the bone defects, the induced membranes were approximated with 0 PDS sutures. As the membranes could not cover all surfaces of the defects, we augmented the coverage the membrane defects with an appropriately sized polypropylene surgical mesh and sutured the mesh to the existing membrane and plate in various areas. The wounds were then closed in layers after placing a drain.

On the first postoperative day, we began knee range of motion exercises and mobilized the patients. Due to articular comminution, patients were instructed to avoid weight-bearing until three months after their initial articular fixation surgery, after which they were encouraged to engage in partial to full weight-bearing.

#### Follow-up

**Fig. 8:**
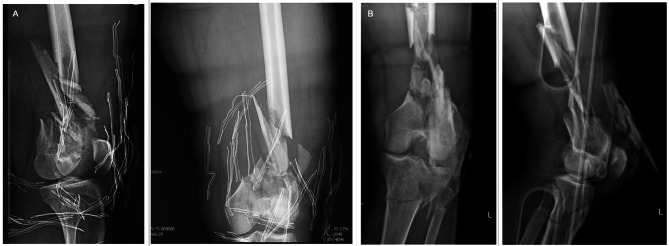
Pre-op X-rays. (**A**) Patient One (**B**) Patient Two

**Fig. 9:**
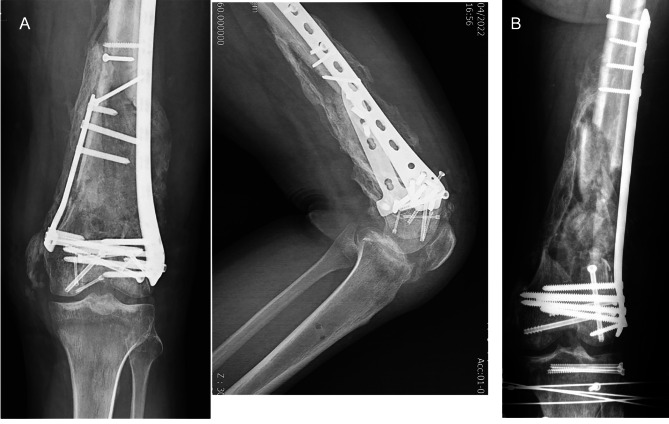
Follow-up X-ray after 3 months of bone grafting in a case with open C3 distal femur fracture. (**A**) Patient One (**B**) Patient Two

During the COVID-19 pandemic, we utilized virtual platforms, primarily the WhatsApp Messenger, to conduct follow-up sessions and instruct physical therapy movements. From the second month after the bone grafting, we started the imaging evaluation for union status and we repeated the x-rays until both clinical and radiological union were seen compared to pre-op x-rays (Figs. 8 and 9). Patients were followed for at least one year and at the final follow-up, we used a validated Lower Extremity Functional Score (LEFS) [2, 34] to assess the function of our patients.

## Results

All five patients’ articular and meta-diaphyseal fractures with bone loss healed without major complications, such as fracture-related infection or nonunion; however, one patient needed heterotopic ossification excision. During their follow-up, none of our patients required any additional surgical procedures for bone healing. From initial trauma to complete radiological and clinical union, the average time was 159 days. Fracture-related infection did not occur in any of our patients in one year of follow-up. After one year of follow-up, the patients’ mean knee range of motion was 5 to 95 degrees. One patient (type 3 C Gustilo case) had knee ligamentous instability with 10-degree genu recurvatum due to Lateral Cruciate Ligament (LCL) and Posterior Cruciate Ligament (PCL) injuries and needed to walk with a hinged knee brace; the patient’s mean Leg length discrepancy (LLD) was 1 cm, as measured clinically. One patient had donor site morbidity on the iliac crest, as well as 3 months of local pain on his iliac wing. No patient experienced any foreign body adverse reactions from the propylene surgical mesh. None of our patients had early post-traumatic osteoarthritis. One patient had developed heterotopic ossification or ectopic bone formation around the knee joint capsule and quadriceps muscle, which limited the joint range of motion by some degrees and it was surgically removed during an outpatient procedure. The average LEFS was 49 out of 80. Findings are summarized in Table 1.

## Discussion

There is currently no consensus on the best management strategy for open distal femoral fractures because of their complexity [27]. The management becomes more difficult in patients with significant bone loss [12]. Although induced membrane procedures are seen to be a proper method for managing large bone defects, they require a stabilizer which has classically been recommended as an intramedullary nail in large bone defects [21]; however, using intramedullary retrograde nailing is not recommended for intra-articular AO/OTA type 33-C3 femoral fractures of the distal femur in several studies [8, 14]. Some reports show that nailing for type C3 is associated with the worse range of motion outcomes among all distal femoral fracture patterns [1]. Management of type C3 with a new locking intramedullary nail is reported as a reliable fixation method [42]; however, it is not a standard of care and the nail is not accessible easily worldwide. An external fixator may also be used for stabilization, but it leads to delayed weight bearing and may lead to knee stiffness and further complications in distal femoral fracture management [5, 20]. However, plating is reported to have favorable outcomes in this regard [13, 14, 37]. Based on the daily living demands, a different range of motion in the knee joint is required [39]; this outcome becomes more important while the patient is involved with high activity levels like sports [3]. For these reasons, we avoided using intra-medullary nailing fixation for any of our cases.

This study found that early fixation of open distal femoral fractures using an anatomical LCP in combination with a modified Masquelet technique along with using propylene surgical mesh resulted in successful clinical and radiographic outcomes. A recent systematic evaluation of 17 studies revealed a significant probability of complication (49.6%) in Masquelet procedures, with a more than 10% non-union rate in cases with bone defect lengths ranging from 0.6 to 26 cm [30]. However, no complications such as nonunion or infection occurred in any of our cases. When compared to previous studies using the plate for fixation, our findings revealed a considerably earlier union time (159 days) [7, 9, 32]. This notable discovery may be related to the hard callus formation found by accident in this study using the modified Masquelet approach. It could also be a call to modify the Masquelet technique in the repair of additional fracture sites to obtain a quicker union and better clinical and radiological outcomes.

The PMMA spacer is inserted to act as a foreign body to induce membrane formation. However, it is not without flaws. The exothermic reaction that occurs during this method’s polymerization damages the bone ends and increases the risk of adjuvant toxicity. In some cases, surgical removal is required, which is difficult to handle because it may cause membrane damage [6, 19]. The arteries and nerves surrounding the bone defect area will not be subjected to heat stress if a substitute material is used that does not cause an exothermic response [35]. Recent studies have attempted to improve the osteogenic properties of the induced membrane by introducing alternative materials [19, 40]. In our case series, we filled the bone defect with shell-shaped antibiotic-impregnated PMMA beads after letting the cement get cold outside the patient’s body [30]. Morelli et al. in their systematic review found that the most common main complications of the Masquelet technique are superficial (4.9%) and deep surgical site infections (4.4%). It is well known that antibiotic beads are used in open fracture reconstructions of the lower extremities [29, 31]. Also, previous studies showed that antibiotics in spacers can help make membranes thicker by increasing osteogenic gene expression and decreasing bacterial inoculation [38], and this is the strength of our technique that we could use the antibiotic beads in our cases, and we believe the use of antibiotic beads in our cases along with meticulous debridement of the open fractures may have contributed to the absence of post-operative infections. We also attempted to widen the cross-section of our beads in order to get a better antibiotic release by molding them into shell-shaped beads. This could potentially be one of the reasons for the absence of infection.

The results of PMMA substitute spacer materials such as titanium have been evaluated in some earlier studies, mostly animal studies; however, low union rates with these rough surfaced spacers have been reported [18]. PMMA substitutes that are less expensive and more easily available can be a good choice for this procedure even in low-income and developing countries; Mathieu et al. published an animal study on rats that used a polypropylene spacer from disposable syringes as a material that met these criteria [25]. In that animal study, it was shown that polypropylene leads to similar tissue reactions as PMMA, with comparable bone healing characteristics probably in terms of growth factors, histology, and stem cell content [25]. To the best of our knowledge, there is only one published clinical study that used polypropylene in Masquelet, which reports two cases of metacarpal bone reconstruction following a gunshot wound using polypropylene syringe body instead of PMMA cement [33]. Polypropylene syringes used in a few previous studies had notable drawbacks. They may not provide the desired stability, and cannot be loaded with antibiotics to prevent infections [24, 25].

In this investigation, we used surgical mesh wrapped around the cement beads to act as a stabilizer as well as a barrier against surrounding tissue irritation. Additionally, we allowed the cement to cool outside the patient’s body, which prevented tissue irritation. Not only did polypropylene mesh function satisfactorily in membrane induction, but it also has substantial other benefits that make it a potential substitute for PMMA, which is traditionally used in the Masquelet technique.

To the best of our knowledge, this is the first clinical study to evaluate the results of the Masquelet technique using polypropylene mesh and PMMA together. This case series, however, is not without limitations. There is no control group observed, and the sample size is small the results presented here may not be generalizable to a larger population. Further research with larger sample size and a control group may shed a light on various aspects of this bone reconstruction method, particularly on the callus formation noted in our study.

## Conclusions

We concluded that comminuted intra-articular open distal femoral fractures with significant bone loss can be managed by primary debridement of devitalized bones, fixation of fracture by anatomical plates and mini fragment screws along with modified Masquelet technique using antibiotic-impregnated PMMA beads augmented by polypropylene mesh in both stages of Masquelet technique. This method can lead to promising outcomes and low complication rates.

## Data Availability

The data used and/or analyzed during the current study are available from the corresponding author on reasonable request.

## List of abbreviations

CRP: C-reactive protein
LCL: Lateral Cruciate Ligament
LCP: Locking compression plate
LEFS: Lower Extremity Functional Score
LLD: Leg length discrepancy
PCL: Posterior Cruciate Ligament
PMMA: Polymethylmethacrylate
